# Deep Learning Can Differentiate IDH-Mutant from IDH-Wild GBM

**DOI:** 10.3390/jpm11040290

**Published:** 2021-04-09

**Authors:** Luca Pasquini, Antonio Napolitano, Emanuela Tagliente, Francesco Dellepiane, Martina Lucignani, Antonello Vidiri, Giulio Ranazzi, Antonella Stoppacciaro, Giulia Moltoni, Matteo Nicolai, Andrea Romano, Alberto Di Napoli, Alessandro Bozzao

**Affiliations:** 1 Neuroradiology Unit, NESMOS Department, Sant’Andrea Hospital, La Sapienza University, Via di Grottarossa 1035, 00189 Rome, Italy; luca.pasquini@uniroma1.it (L.P.); francesco.dellepiane@uniroma1.it (F.D.); giulia.moltoni@uniroma1.it (G.M.); matteo.nicolai@uniroma1.it (M.N.); andrea.romano@uniroma1.it (A.R.); alberto.dinapoli@uniroma1.it (A.D.N.); alessandro.bozzao@uniroma1.it (A.B.); 2 Neuroradiology Service, Department of Radiology, Memorial Sloan Kettering Cancer Center, 1275 York Ave, New York, NY 10065, USA; 3 Medical Physics Department, Bambino Gesù Children’s Hospital, IRCCS, Piazza di Sant’Onofrio, 4, 00165 Rome, Italy; emanuela.tagliente@opbg.net (E.T.); martina.lucignani@opbg.net (M.L.); 4 Radiology and Diagnostic Imaging Department, Regina Elena National Cancer Institute, IRCCS, Via Elio Chianesi 53, 00144 Rome, Italy; antonello.vidiri@ifo.gov.it; 5 Surgical Pathology Unit, Department of Clinical and Molecular Medicine, Sant’Andrea Hospital, La Sapienza University, Via di Grottarossa 1035, 00189 Rome, Italy; giulio.ranazzi@uniroma1.it (G.R.); Antonella.stoppacciaro@uniroma1.it (A.S.)

**Keywords:** GBM, high grade glioma, MRI, IDH, deep learning, CBV, artificial intelligence

## Abstract

Isocitrate dehydrogenase (IDH) mutant and wildtype glioblastoma multiforme (GBM) often show overlapping features on magnetic resonance imaging (MRI), representing a diagnostic challenge. Deep learning showed promising results for IDH identification in mixed low/high grade glioma populations; however, a GBM-specific model is still lacking in the literature. Our aim was to develop a GBM-tailored deep-learning model for IDH prediction by applying convoluted neural networks (CNN) on multiparametric MRI. We selected 100 adult patients with pathologically demonstrated WHO grade IV gliomas and IDH testing. MRI sequences included: MPRAGE, T1, T2, FLAIR, rCBV and ADC. The model consisted of a 4-block 2D CNN, applied to each MRI sequence. Probability of IDH mutation was obtained from the last dense layer of a softmax activation function. Model performance was evaluated in the test cohort considering categorical cross-entropy loss (CCEL) and accuracy. Calculated performance was: rCBV (accuracy 83%, CCEL 0.64), T1 (accuracy 77%, CCEL 1.4), FLAIR (accuracy 77%, CCEL 1.98), T2 (accuracy 67%, CCEL 2.41), MPRAGE (accuracy 66%, CCEL 2.55). Lower performance was achieved on ADC maps. We present a GBM-specific deep-learning model for IDH mutation prediction, with a maximal accuracy of 83% on rCBV maps. Highest predictivity achieved on perfusion images possibly reflects the known link between IDH and neoangiogenesis through the hypoxia inducible factor.

## 1. Introduction

Glioblastoma multiforme (GBM) is the most lethal primary brain tumor for an adult and accounts for 70–75% of all diffuse gliomas [1] and 16% of primary central nervous system (CNS) tumors in adults [2]. Despite the state-of-the-art treatment of implementing a combination of temozolomide and tailored radiotherapy, overall survival is very limited, with a median between 14 and 17 months [1]. Genetic profile directly impacts diagnosis and therapy of gliomas, with demonstrated effects on survival [3]. One of the most important genetic biomarkers of GBM is isocitrate dehydrogenase (IDH) [4]. Most GBMs are primary (90%) and rarely harbor an IDH mutation (3.7%) [4]. Secondary GBMs represent around 10% of total cases and are more likely to be mutated (73%) [4]. Overall, IDH mutation is expected in about 10% of GBMs [5]. Most importantly, mutant GBMs are characterized by significantly improved survival rather than wild-type GBMs (31 months vs. 15 months) [3,4,6,7,8]. In addition, patients with IDH1-mutated glioblastomas show better outcomes than IDH1 wild-type gliomas of a lower grade [9]. These considerations inspired the cIMPACT recommendations for classification of diffused gliomas, which suggested that considering IDH-mutant and IDH-wild type GBM as two separate entities due to the importance of IDH mutation for patient survival [10].

The gold-standard procedure for the diagnosis of GBM is pathological sampling through brain biopsy or surgery. Along with the risk of complications, high costs and misinterpretation rates, biopsy-based methods may face incomplete sampling due to spatial heterogeneity of GBMs, characterized by multiple intra-tumoral habitats and variable genetic expressions [11,12]. Also, various studies reported limitations for IDH mutation pathological testing due to technical shortcomings [13,14,15], supporting the need for non-invasive diagnostic procedures to complement the ground truth of pathologic evidence. Magnetic resonance imaging (MRI) is a versatile examination that can provide a non-invasive definition of genetic alterations in the pre-operative setting, guide targeted biopsies, screen highly probable genetic mutations for further testing and help tailoring treatment interventions to the single case [16].

**Figure 1:**
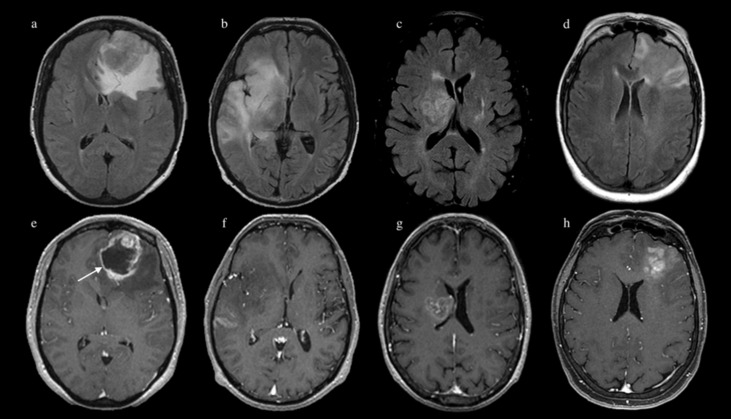
FLAIR images (above) and post-contrast MPRAGE images (below) of four patients with glioblastoma multiforme (GBM) from our cohort. Patient 1 (**a**,**e**) presented an expansile left frontal lobe GBM with typical features of rim-enhancement and central necrosis (arrow on image e); pathology confirmed isocitrate dehydrogenase (IDH)-wildtype. Patient 2 (**b**,**f**) presented a diffuse non-enhancing infiltrative GBM of the right temporo-insular region; pathology confirmed IDH mutation. Patient 3 (**c**,**g**) and 4 (**d**,**h**) demonstrated ‘borderline’ features, not typical for any IDH status; pathology confirmed IDH mutation for patient 3 and IDH-wild type for patient 4.

Despite the importance for patient outcome and treatment, IDH-mutation does not present a clear radiologic signature [5]. For example, IDH mutant gliomas may show less enhancement, less blood flow on perfusion-weighted images, higher mean diffusion values, smaller sizes and frontal lobe location [17]. However, the sensitivity of these findings is somehow disappointing in GBM [5,18] (Figure 1).

In the last few years, the implementation of artificial intelligence in the field of radiomics held a great advancement in our understanding of the correlations between radiological features and genetic phenotypes [16]. Supervised machine learning (ML) techniques achieved good performance in predicting IDH mutation in GBM [17]. However, these techniques are time consuming and require expert supervision, being far from clinical implementation. Deep learning may offer a solution to these shortcomings, thanks to the ability of independently learning the features that are most relevant for the task, without the constraint of a priori selection [19]. Previous studies based on this approach mostly focused on differentiating IDH status in heterogeneous populations of low-grade gliomas (LGG) and high-grade gliomas (HGG) [19]. A GBM-specific model for IDH prediction is lacking evidence in literature, despite being highly relevant due to paucity of non-invasive biomarkers.

Our objective was to build a reliable deep-learning model predictive of IDH mutation in patients with GBM, starting from conventional and advanced MRI data.

## 2. Materials and Methods

### 2.1. Subjects

This retrospective observational study was conducted in agreement with the Helsinki declaration and was approved by the Intitutional Review Board (IRB) (protocol number: 19 SA_2020). We enrolled patients who underwent preoperative MRI from March 2005 to May 2019, fulfilling the following inclusion criteria: histopathological diagnosis of GBM, MRI acquisition in the preoperative phase with at least one among structural, diffusion or perfusion techniques, and IDH testing. Exclusion criteria were motion artifacts or other causes of sub-optimal images.

### 2.2. Histopathological Analysis

The specimens were fixed in 10% formaldehyde and processed for paraffin embedding. Two-micrometer-thick sections were mounted and stained with hematoxylin and eosin. Histopathological examination, typing and grading were performed identifying at least three of the following features in astrocytic tumors: mitotic figures, cellular atypia, microvascular proliferation and/or necrosis, according to the last edition of the World Health Organization classification of CNS tumors.

Immunohistochemistry was performed using Dako Envision Flex system. For the evaluation of IDH-1 mutation, IDH-1 R132H antibody was used. The test was defined as positive if a focal or diffuse immunopositivity was detected and negative if no tumor cells showed immunopositivity. Negative cases were then analyzed for IDH-1/2 mutations by directly sequencing exon 4 of the IDH1 gene including codon 132, and a fragment of 219 bp in length spanning the catalytic domain of *IDH2* including codon 172 following PCR amplification of genomic DNA using respectively the primers IDH-1: forward, 5′-CGG TCT TCA GAG AAG CCA TT-3′, reverse, 5′- ATT CTT ATC TTT TGG TAT CTA CAC C-3′, IDH-2: forward 5′-CAAGCTGAAGAAGATGTGGAA-3′, reverse 5′ CAGAGACAAGAGGATGGCTA-3′. All sequence reactions were carried out using the GenomeLab DTCS quick-start kit (Beckman Coulter, Fullerton, CA, USA). The reactions were carried out in an automated DNA analyzer (CEQ 8000; Beckman Coulter, Fullerton, CA, USA).

### 2.3. MR Image Acquisition

Acquired MR sequences included MPRAGE, FLAIR, T1-weighted, T2-weigthed, diffusion weighted images (DWI), with apparent diffusion coefficient (ADC) map reconstruction, and perfusion weighted images (PWI) with dynamic susceptibility contrast (DSC) technique. MRI examinations were acquired on a 1.5 T (Magnetom Sonata, Siemens, Erlangen, Germany) and a 3 T scanner (Discovery MR750w; GE Medical Systems, Waukesha, WI, USA). Patients underwent the following protocol: axial T1-weighted spin echo, axial T2-weighted fast spin echo, axial FLAIR; DWI acquired with three levels of diffusion sensitization (*b*-values 0, 500 and 1000); DSC acquired during contrast injection (DOTAREM.; dose 0.1 mmol/kg, injection rate 4 mL/s), followed by a 20-mL saline flush, based on T2*-weighted gradient-echo echo-planar; MPRAGE after administration of contrast. Perfusion parametric maps were obtained through a dedicated software package OleaSphere software version 3.0 (Olea Medical, La Ciotat, France). A rCBV map was generated by using an established tracer kinetic model applied to the first-pass data [20]. As previously described [21], the dynamic curves were mathematically corrected to reduce contrast agent leakage effects.

### 2.4. Image Processing

All MR images were co-registered to an individual MPRAGE sequence. In order to normalize pixels intensity, the tumor lesion area was derived by a bounding box function implemented on Python3. Data were masked according to a region of interest (ROI) obtained from T2 images in order to obtain the pixels of the whole tumor region, including peritumoral edema. All the other pixels were set to zero and the mean of the tumor region was used for scaling the entire volume data. Images intensities were normalized by subtracting the median intensity of the entire brain and dividing by the standard deviation of the same portion, as used in previous work [22]. For each patient, the axial slice with the widest tumor extension was selected, and a bounding rectangle derived from the tumor mask was drawn around the tumor. As each tumor was different in size, all images were resampled to 64 × 64 [23,24]. Since not all MRI sequences were acquired for every patient and some patients lacked full genetic testing, the dataset was subject to imbalance issues. To minimize overfitting, data augmentation techniques were employed [24,25,26]. The number of samples for each test is reported in Table 1.

As the dataset under investigation included GBM patients only, IDH labels were unbalanced towards wildtype tumors [4]; for this reason, a threshold of at least 5 IDH-mutant patients was set for the testing group during randomization.

Once the dataset was split into training and testing sets (test_size = 20%), augmentation allowed to fix the unbalance between classes in the training set. The augmentation method Image Data Generator class (Keras API) was employed with rotation_range = [0°,90°]. Other geometric transformations influencing tumor shape (such as flipping, color space, cropping, translation, noise injection) [27] were excluded as they might interfere with the final prediction accuracy, since shape features correlate to IDH mutation in our experience [28].

### 2.5. CNN Architecture

**Figure 2:**
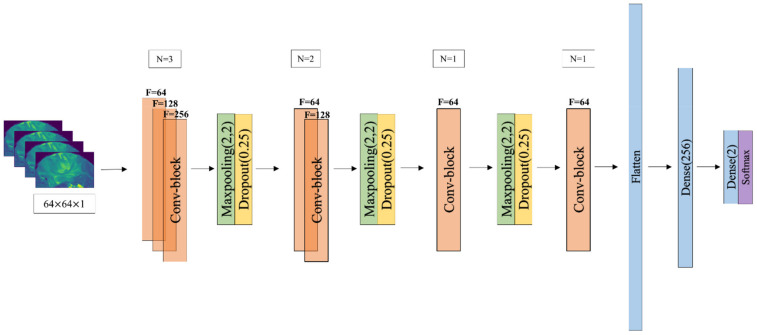
Proposed convoluted neural networks (CNN) architecture to predict IDH status. N = number of Conv-2D operations; F = number of trainable filters.

We propose a 2D CNN model with a set of 2D trainable filters. CNN derives high-level features from the low-level input, while estimated high-level features directly contribute to the classification of input data. The network architecture usually consists of a number of layers, which generate progressively higher-level features as we go deeper into the network. Inspired by very deep convolutional networks (e.g., VGGNet, ResNet), we designed a 4-block 2D CNN architecture (Figure 2). Rectified linear layer (ReLU) and batch normalization, both after the 2D convolutional layer, compose the convolutional block. The CNN input was a 64 × 64 2D MR slice extracted at the level of maximal tumor extension. The convolutional layers computed their outputs from the input slice by applying convolutional operations with 3 × 3 2D filters. The result was an output feature map the same size as the input, followed by max-pooling to down-sample the image. Downstream from the convolutional blocks, the last three CNN layers were fully connected. Particularly, following the last convolutional block, a fully connected layer preceded the last two neurons of the output. This layer was responsible for assessing classification probabilities for IDH-wild type versus IDH-mutant GBMs. IDH mutation probability was calculated with the softmax algorithm for each sample, and categorical cross-entropy loss (CCEL) was chosen as objective function of our network with two output nodes. We employed 5-fold cross-validation, so that the performance measures average over five different splitting of training and testing data, keeping a minimum threshold of 5 IDH-mutant patients in the testing cohort. To demonstrate the individual predictive performance of different MRI sequences, T1-weighted-, T2-weighted-, FLAIR-, MPRAGE-, ADC-, and rCBV-fed networks were trained for 500 epochs separately. Each epoch takes 7 s to iterate on the entire training dataset, with a complex training time of 1 h for each MRI sequence. As previously employed in a very similar study [29], Adam with a learning rate of 0.0001 was chosen as optimizer due to higher speed in reaching convergence in deep CNNs [30]. Model training was performed on NVIDIA-SMI GPU (CUDA Version, NVIDIA, Beijing, China). Our pipeline was written in Python3, using the Keras API Framework (https://keras.io/about/ accessed on 5 March 2020).

## 3. Results

We selected 156 adult patients (mean age = 62 y, range = 35–83 y) with pathologically-proven diagnosis of GBM. Among these patients, 100 underwent IDH testing and were included in the study. Histopathological analysis demonstrated 83 IDH-wild-type (42 males; mean age 63 years; mean overall survival 491 days; MGMT promoter methylation status: 55% methylated, 35% unmethylated, 8% unknown) and 17 IDH-mutant GBM (11 males; mean age 56 years; mean overall survival 613 days; MGMT promoter methylation status: 77% methylated, 23% unmethylated). All patients received postoperative focal radiotherapy plus concomitant daily temozolomide (TMZ), followed by adjuvant TMZ therapy, with the same treatment protocol.

**Figure 3:**
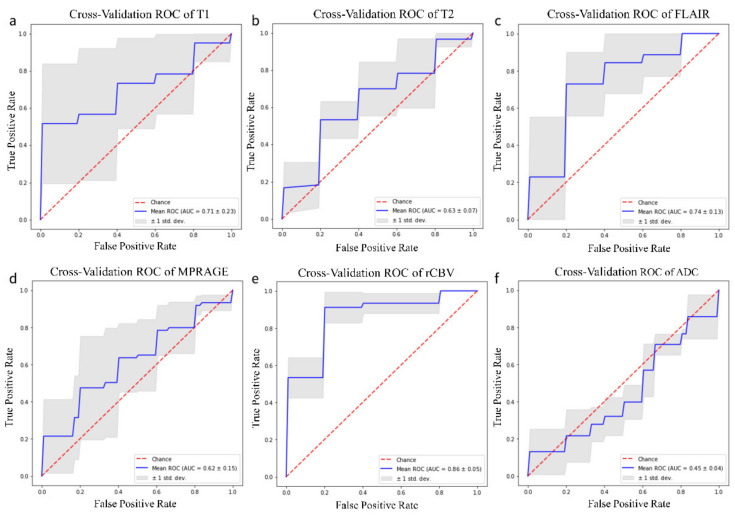
ROC curve for testing set from k-fold cross validation (k = 5) training on T1-weighted magnetic resonance imaging (MRI) sequence (**a**), T2-weighted MRI sequence (**b**), FLAIR MRI sequence (**c**), MPRAGE MRI sequence (**d**), rCBV MRI sequence (**e**), ADC MRI sequence (**f**).

IDH mutation prediction performance was averaged over five different splitting of training and testing data. The final model for T1-weighted, T2-weighted, FLAIR, MPRAGE, rCBV and ADC images achieved mean AUCs of 0.71, 0.63, 0.74, 0.62, 0.86, 0.45 respectively. ROC curves are reported in Figure 3 for every MR sequence.

Accuracy, loss, sensitivity and specificity on the independent testing cohort were as follows: 77%, 1.4, 36%, 95% for T1-weighted sequence; 67%, 2.41, 48%, 75% for T2-weighted sequence; 77%, 1.98, 28%,95% for FLAIR sequence; 66%, 2.55, 43%, 74% for MPRAGE sequence; 83%, 0.64, 76%, 86% for rCBV sequence and 56%, 2.53, 14%, 73% for ADC.

**Figure 4:**
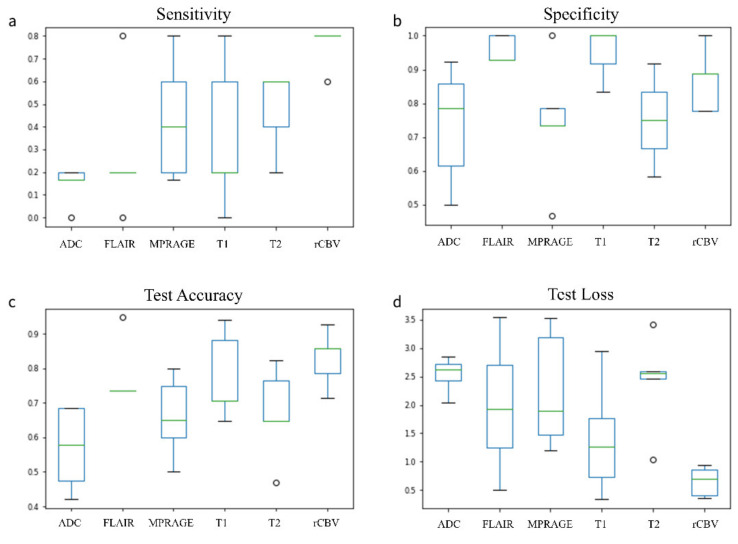
Boxplot on k-fold cross validation (k = 5) for sensitivity (**a**), specificity (**b**), test accuracy (**c**) and test loss (**d**) with all MRI sequences.

Predictive performances are reported in Table 2, with corresponding boxplots in Figure 4. In the Supplementary Materials, we reported learning plots fitted with an exponential function (1) for the testing cohort:

(1) $$f\left(x\right)=a\times e^{-b\times x}+c$$

## 4. Discussion

**Figure 5:**
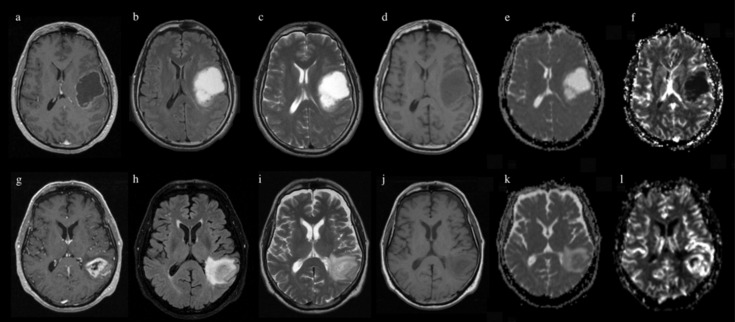
Examples of correctly predicted outcome (IDH mutation) from our CNN model. Images above show MPRAGE (**a**), FLAIR (**b**), T2 (**c**), T1 (**d**), ADC (**e**) and rCBV (**f**) sequences of a patient with IDH mutated GBM. Images below show MPRAGE (**g**), FLAIR (**h**), T2 (**i**), T1 (**j**) ADC (**k**) and rCBV (**l**) sequences of a patient with IDH-wild-type GBM.

**Figure 6:**
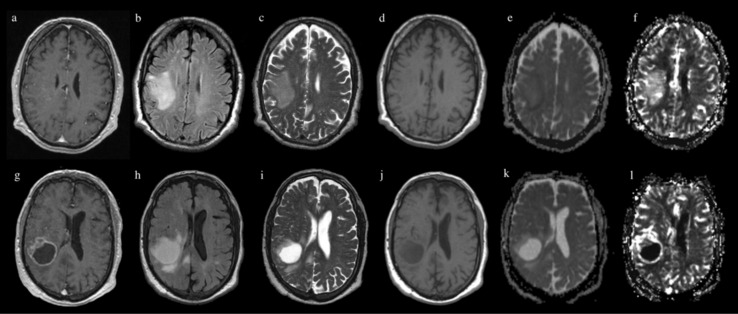
Examples of incorrectly predicted outcome (IDH mutation) from our CNN model. Images above show MPRAGE (**a**), FLAIR (**b**), T2 (**c**), T1 (**d**), ADC (**e**) and rCBV (**f**) sequences of a patient with IDH mutated GBM predicted as wild type (false negative). Images below show MPRAGE (**g**), FLAIR (**h**), T2 (**i**), T1 (**j**) ADC (**k**) and rCBV (**l**) sequences of a patient with IDH-wild-type GBM predicted as mutated (false positive).

This is the first study to attempt IDH status prediction in GBM. Our model achieved a good prediction performance for IDH genotype, especially on rCBV maps (83% accuracy, 76% sensitivity, 86% specificity), showing comparable or superior results to other studies employing CNN architectures on gliomas [23,25,26,31]. Examples of predicted outcome (IDH status) are reported in Figure 5 and Figure 6.

Mutations of IDH coding gene lead to accumulation of D-2 hydroxyglutarate (D-2HG), an oncogenic metabolite which may affect cellular differentiation [7,8,32]. This reflects on tumoral features, such as cellularity, pattern of growth, vascularization, which presents a radiologic correlate on MRI [33,34]. Even though the human eye may not capture these features with acceptable accuracy [5,18], modern artificial intelligence techniques can help in overcoming these limitations. Radiomics achieved remarkable results in providing biomarkers for patient survival and tumor genetics [16]. For example, Hsieh et al. achieved an accuracy of 85%, a sensitivity of 86%, and a specificity of 84% in predicting IDH mutation in GBM with texture features and a binary logistic regressor classifier [35]. Zhang et al. obtained 89% accuracy in predicting IDH mutation in HGG with a random forest classifier [36]. Both of these studies relied on supervised ML algorithms for the prediction tasks, which are not exempted from shortcomings: lack of parameter standardization may limit reproducibility and reliability of these models [37]. Our group recently investigated the performance of several ML classifiers with different optimization parameters, highlighting how ensemble architectures show better results in clinical tasks prediction for GBM [28]. However, this standard radiomic workflow retains several steps with supervision constraints, such as feature extraction and selection [38,39].

In recent years, deep learning emerged as a promising technique to analyze imaging data [19]. Deep learning employs specific architectures named CNNs to achieve task prediction without human supervision [40]. Regarding our topic, Chang et al. predicted IDH mutation in a group of tumors including LGG and HGG by employing a 2D residual CNN (ResNet34) with 82.8% accuracy for training, 85.7% for testing and 83.6% for validation cohorts [25]. Starting from this architecture, Liang et al. implemented a Multimodal 3D DenseNet for IDH prediction, achieving 84.6% accuracy, which was boosted to 91.4% when associated with transfer learning [26]. Other authors [31] described a CNN for prediction of IDH mutation, MGMT methylation, and 1p/19q co-deletion from 256 brain MRIs from the Cancer Imaging Archives Dataset including LGG and HGG. They reported 94% accuracy for IDH, 92% for 1p/19q, and 83% for MGMT status. These studies rely on heterogeneous glioma populations [25,26,31] which may represent a bias when such results are applied to GBM alone. For example, since IDH-mutant GBMs are very rare, identification of IDH mutation may simply reflect tumor grade in a heterogeneous glioma group, especially when automatic predictive models are employed. Indeed, LGGs show typical radiologic features: less enhancement, less necrotic components and a diffused pattern of growth [5]. Differently, most GBMs show peripheral enhancement and central necrosis on MRI, with a surrounding admixture of infiltrative tumor and vasogenic edema [5] (Figure 1). Chang et al. applied principal component analysis to the final output layer of their CNN to provide a visualization of the top-ranking features associated with IDH mutation [31]. As result, T1 post-contrast features demonstrated the highest rank: IDH-wildtypes showed thick and irregular enhancement or irregular, ill-defined, peripheral enhancement, while IDH-mutants displayed less enhancement and well-defined tumor margins. This result seems to capture the radiologic appearance of HGG vs. LGG more than IDH-specific features [5].

Predicting IDH mutation in GBM is extremely important since conventional imaging features of IDH mutated tumors show considerable overlap with those of IDH-wildtypes [18] (Figure 1). In addition, primary and secondary GBMs are generally indistinguishable at standard MRI, although secondary GBM harbors most of IDH mutations [5]. Our study proposes a new deep learning model tailored to GBM, with high prediction performance for IDH status on MRI sequences. This has several advantages, including the automatic pipeline of our model limits high-skilled programming with easy handling for further applications. Moreover, our study is the first to explore the predictive power of advanced MRI sequences such as DWI and DSC perfusion.

The high IDH prediction accuracy obtained with perfusion images is consistent with previous studies [41,42]. Besides reflecting tumor grade [43], perfusion parameters showed a promising correlation with patient survival, particularly rCBV [44]. In our experience, we achieved high predictivity of GBM IDH status with machine ML on rCBV data [28]. Kieckegereder et al. demonstrated that *IDH* mutation status is associated with a specific hypoxia/angiogenesis transcriptome signature through perfusion imaging [41]. Wu et al. extracted GBMs vascular habitats based on DSC perfusion, reporting that IDH mutation correlates to rCBV values of the low-angiogenic habitat from the enhancing tumor. These results reflect the known link between IDH mutation and neoangiogenesis through the hypoxia inducible factor [45]. Our CNN architecture computes automated deep features from input images, extracting semantic regional features including not only the tumor core but also the peritumoral edema as we chose to include the entire tumor from T2-ROI masks (see Methods). A study from Chow et al. may support our choice, revealing that GBM induces vascular dysregulation in peritumoral regions, which are larger in IDH-wildtype than in IDH-mutant gliomas, helping to differentiate IDH genotypes [46]. One may argue that diffusion parameters were expected to achieve higher performance, since ADC correlates to tumor cellularity [47,48] and showed good results in predicting patient survival [49]. However, radiogenomic associations with IDH are poorly explored in GBM [17]. Also, diffusion parameters may fail to capture differences in a relatively homogeneous GBM group.

Our study has some limitations. First of all, the number of subjects may be considered small for deep-learning applications. We employed data augmentation (see methods) to partially compensate this issue. Due to the limited samples in our dataset, in order to prevent overfitting, we reduced the number of learning parameters generated by each layer. This prevented us from employing other architectures, such as ResNet, which generate a large number of parameters, making the training unfeasible. Additionally, our analysis relied on two cohorts due only to population size. We performed cross-validation to overcome the problem of randomization and the lack of a validation set. Differently from previous studies, we could not include patients from public datasets because of lack of perfusion images.

## 5. Conclusions

Our deep-learning model achieved a maximal accuracy of 83% on rCBV images, in agreement with previous studies showing a correlation between IDH mutation and angiogenesis in gliomas. Lower performance achieved on other sequences reflects the known radiological challenge of discriminating IDH status based on anatomical images, as well as a possible failure of diffusion parameters to capture subtle differences in a relatively homogeneous GBM group. Our GBM-specific model leads to several advantages to non-invasively estimate IDH mutation, disentangling the prediction from WHO grade.

## Figures and Tables

**Table 1:** Overview of patient data before and after data augmentation.

Sequence	Subjects Intersection	Train/Test (Split = 0.2)	AugTrain/Test	Train:0/1	Test:0/1
T1	83	65/17	106/17	53/53	5/12
T2	84	66/17	108/17	54/54	5/14
FLAIR	96	76/19	124/19	62/62	5/14
MPRAGE	97	76/20	128/20	64/64	5/15
rCBV	66	52/14	90/14	45/45	5/9
ADC	93	73/19	124/19	62/62	5/14

Subjects intersection = effective number of subject with IDH label; train/test (split = 0.2) = number of samples for train and test with 20% split and no augmentation; AugTrain/test = number of augmented train samples and test samples; train:0/1 = balanced number of samples thanks to augmentation; test:0/1 = number of samples with class 0 vs. class 1 for the test group.

**Table 2:** Predictive performance of each MR sequence indicated as mean ± standard deviation.

Sequence	Test Acc	Test Loss	SN	SP	AUC
T1	0.77 ± 0.11	1.4± 09	0.36 ± 0.29	0.95 ± 0.07	0.71 ± 0.23
T2	0.67 ± 0.12	2.41 ± 0.77	0.48 ± 0.16	0.75 ± 0.12	0.63 ± 0.07
FLAIR	0.77 ± 0.11	1.98 ± 1.06	0.28 ± 0.27	0.95 ± 0.03	0.74 ± 0.13
MPRAGE	0.66 ± 0.1	2.55 ± 0.93	0.43 ± 0.24	0.74 ± 0.17	0.62 ± 0.15
rCBV	0.83 ± 0.07	0.64 ± 0.23	0.76 ± 0.08	0.86 ± 0.08	0.86 ± 0.05
ADC	0.56 ± 0.11	2.53 ± 0.28	0.14 ± 0.07	0.73 ± 0.16	0.45 ± 0.04

Acc = accuracy; SN = sensitivity; SP = specificity; AUC = area under the curve.

## Data Availability

The data presented in this study are available on request from the corresponding author.

## Supplementary Materials

The following are available online at https://www.mdpi.com/article/10.3390/jpm11040290/s1, Figure S1: Test accuracy and test loss learning plot for T1-weighted MRI sequence, according to five different dataset splitting randomizations (R1, R2, R3, R4, R5), compared with the training accuracy and loss learning plot (blue line). The dotted lines in red show the mean above all test accuracy (up) and loss curves (down), Figure S2: Test accuracy and test loss learning plot for T2-weighted MRI sequence, according to five different dataset splitting randomizations (R1, R2, R3, R4, R5), compared with the training accuracy and loss learning plot (blue line). The dotted lines in red show the mean above all test accuracy and loss curves, Figure S3: Test accuracy and test loss learning plot for FLAIR sequence, according to five different dataset splitting randomizations (R1, R2, R3, R4, R5), compared with the training accuracy and loss learning plot (blue line). The dotted lines in red show the mean above all test accuracy and loss curves, Figure S4: Test accuracy and test loss learning plot for MPRAGE MRI sequence, according to five different dataset splitting randomizations (R1, R2, R3, R4, R5), compared with the training accuracy and loss learning plot (blue line). The dotted lines in red show the mean above all test accuracy and loss curves, Figure S5: Test accuracy and test loss learning plot for rCBV MRI sequence, according to five different dataset splitting randomizations (R1, R2, R3, R4, R5), compared with the training accuracy and loss learning plot (blue line). The dotted lines in red show the mean above all test accuracy and loss curves, Figure S6: Test accuracy and test loss learning plot for ADC MRI sequence, according to five different dataset splitting randomizations (R1, R2, R3, R4, R5), compared with the training accuracy and loss learning plot (blue line). The dotted lines in red show the mean above all test accuracy and loss curves, Table S1: Demographic and molecular data for training and testing cohorts with rCBV sequence, Table S2: Demographic and molecular data for training and testing cohorts with T1 and T2 sequences, Table S3: Demographic and molecular data for training and testing cohorts with ADC sequence, Table S4: Demographic and molecular data for training and testing cohorts with FLAIR sequence, Table S5: Demographic and molecular data for training and testing cohorts with MPRAGE sequence.
