# Supplementary material for: Deep Learning Can Differentiate IDH-Mutant from IDH-Wild GBM
Source: J Pers Med. 2021 Apr 9;11(4):290. doi: 10.3390/jpm11040290 (PMC8069494; doi:10.3390/jpm11040290)
Supplement: Supplementary file 1 [file jpm-11-00290-s001.zip › Captures.docx]

***Figure 1.*** *Test accuracy and test loss learning plot for T1-weighted MRI sequence, according to five different dataset splitting randomizations (****R1, R2, R3, R4, R5****), compared with the training accuracy and loss learning plot (****blue line****). The dotted lines in red show the mean above all test accuracy (up) and loss curves (down).*

***Figure 2.*** *Test accuracy and test loss learning plot for T2-weighted MRI sequence, according to five different dataset splitting randomizations (R1, R2, R3, R4, R5), compared with the training accuracy and loss learning plot (****blue line****). The dotted lines in red show the mean above all test accuracy and loss curves.*

***Figure 3.*** *Test accuracy and test loss learning plot for FLAIR sequence, according to five different dataset splitting randomizations (R1, R2, R3, R4, R5), compared with the training accuracy and loss learning plot (****blue line****). The dotted lines in red show the mean above all test accuracy and loss curves.*

***Figure 4.*** *Test accuracy and test loss learning plot for MPRAGE MRI sequence, according to five different dataset splitting randomizations (R1, R2, R3, R4, R5), compared with the training accuracy and loss learning plot (****blue line****). The dotted lines in red show the mean above all test accuracy and loss curves.*

***Figure 5.*** *Test accuracy and test loss learning plot for rCBV MRI sequence, according to five different dataset splitting randomizations (R1, R2, R3, R4, R5), compared with the training accuracy and loss learning plot (****blue line****). The dotted lines in red show the mean above all test accuracy and loss curves.*

***Figure 6.*** *Test accuracy and test loss learning plot for ADC MRI sequence, according to five different dataset splitting randomizations (R1, R2, R3, R4, R5), compared with the training accuracy and loss learning plot (****blue line****). The dotted lines in red show the mean above all test accuracy and loss curves.*
