# Supplementary material for: Deep Learning Can Differentiate IDH-Mutant from IDH-Wild GBM
Source: J Pers Med. 2021 Apr 9;11(4):290. doi: 10.3390/jpm11040290 (PMC8069494; doi:10.3390/jpm11040290)
Supplement: Supplementary file 1 [file jpm-11-00290-s001.zip › supp_tables.docx]

Supplementary Table 1. Demographic and molecular data for training and testing cohorts with rCBV sequence

| **rCBV** | | |
| --- | --- | --- |
| **Total male\|female** | 27\|25 | 10\|4 |
| **Group (N° patients)** | **Train (n = 52)** | **Test (n = 14)** |
| **Age (average, range)** | 63,(37,80) | 55,(34,78) |
| **Surv < 1 year** | 30 | 5 |
| **Surv > 1 year** | 22 | 9 |
| **Surv days (average, range)** | 400,(40,1757) | 356,(68,600) |
| **KI67 (Label 1/Label 0)** | 33\|11 | 10\|1 |
| **MGMT (Label 1/Label 0)** | 28\|21 | 9\|4 |
| **IDH (Label 1/Label 0)** | 5\|47 | 5\|9 |
| **P53 (Label 1/Label 0)** | 11\|24 | 5\|7 |
| **EGFR (Label 1/Label 0)** | 19\|11 | 5\|2 |

Surv = Overall Survival; KI67 (0=<20%, 1=>20%); MGMT (0=unmethylated, 1=methylated); IDH (0=wild-type, 1=mutated); P53 (0=<30%, 1=>30%); EGFR (0=negative, 1=positive)

Supplementary Table 2. Demographic and molecular data for training and testing cohorts with T1 and T2 sequences

| **T1/T2** | | |
| --- | --- | --- |
| **Total male\|female** | 37\|27 | 8\|9 |
| **Group (N° patients)** | **Train (n = 64)** | **Test (n = 17)** |
| **Age (average, range)** | 62,(34,80) | 63,(35,80) |
| **Surv < 1 year** | 38 | 5 |
| **Surv > 1 year** | 27 | 12 |
| **Surv days (average, range)** | 369,(40,2496) | 527,(40,1427) |
| **KI67 (Label 1/Label 0)** | 43\|8 | 9\|4 |
| **MGMT (Label 1/Label 0)** | 38\|23 | 9\|7 |
| **IDH (Label 1/Label 0)** | 11\|54 | 4\|13 |
| **P53 (Label 1/Label 0)** | 17\|28 | 2\|8 |
| **EGFR (Label 1/Label 0)** | 23\|17 | 6\|0 |

Surv = Overall Survival; KI67 (0=<20%, 1=>20%); MGMT (0=unmethylated, 1=methylated); IDH (0=wild-type, 1=mutated); P53 (0=<30%, 1=>30%); EGFR (0=negative, 1=positive)

Supplementary Table 3. Demographic and molecular data for training and testing cohorts with ADC sequence

| **ADC** | | |
| --- | --- | --- |
| **Total male\|female** | 40\|33 | 11\|8 |
| **Group (N° patients)** | **Train (n = 73)** | **Test (n = 19)** |
| **Age (average, range)** | 62,(34,81) | 63,(35,80) |
| **Surv < 1 year** | 37 | 10 |
| **Surv > 1 year** | 36 | 9 |
| **Surv days (average, range)** | 392,(40,2496) | 443,(102,1757) |
| **KI67 (Label 1/Label 0)** | 43\|11 | 11\|3 |
| **MGMT (Label 1/Label 0)** | 43\|24 | 13\|6 |
| **IDH (Label 1/Label 0)** | 14\|59 | 3\|16 |
| **P53 (Label 1/Label 0)** | 16\|28 | 4\|9 |
| **EGFR (Label 1/Label 0)** | 24\|11 | 5\|7 |

Surv = Overall Survival; KI67 (0=<20%, 1=>20%); MGMT (0=unmethylated, 1=methylated); IDH (0=wild-type, 1=mutated); P53 (0=<30%, 1=>30%); EGFR (0=negative, 1=positive)

Supplementary Table 4. Demographic and molecular data for training and testing cohorts with FLAIR sequence

| **FLAIR** | | |
| --- | --- | --- |
| **Total male\|female** | 39\|37 | 13\|5 |
| **Group (N° patients)** | **Train (n = 76)** | **Test (n = 18)** |
| **Age (average, range)** | 62,(34,81) | 63,(37,80) |
| **Surv < 1 year** | 34 | 13 |
| **Surv > 1 year** | 42 | 5 |
| **Surv days (average, range)** | 444,(40,2496) | 298,(40,1430) |
| **KI67 (Label 1/Label 0)** | 42\|12 | 12\|3 |
| **MGMT (Label 1/Label 0)** | 44\|28 | 13\|3 |
| **IDH (Label 1/Label 0)** | 12\|64 | 3\|15 |
| **P53 (Label 1/Label 0)** | 18\|27 | 3\|11 |
| **EGFR (Label 1/Label 0)** | 25\|14 | 4\|4 |

Surv = Overall Survival; KI67 (0=<20%, 1=>20%); MGMT (0=unmethylated, 1=methylated); IDH (0=wild-type, 1=mutated); P53 (0=<30%, 1=>30%); EGFR (0=negative, 1=positive)

Supplementary Table 5. Demographic and molecular data for training and testing cohorts with MPRAGE sequence

| **MPRAGE** | | |
| --- | --- | --- |
| **Total male\|female** | 41\|35 | 12\|8 |
| **Group (N° patients)** | **Train (n =76)** | **Test (n = 20)** |
| **Age (average, range)** | 63,(34,80) | 60,(37,80) |
| **Surv < 1 year** | 37 | 11 |
| **Surv > 1 year** | 39 | 9 |
| **Surv days (average, range)** | 397,(40,1757) | 503,(40,2496) |
| **KI67 (Label 1/Label 0)** | 44\|13 | 11\|3 |
| **MGMT (Label 1/Label 0)** | 47\|25 | 12\|6 |
| **IDH (Label 1/Label 0)** | 12\|64 | 3\|17 |
| **P53 (Label 1/Label 0)** | 16\|34 | 5\|5 |
| **EGFR (Label 1/Label 0)** | 24\|15 | 6\|3 |

Surv = Overall Survival; KI67 (0=<20%, 1=>20%); MGMT (0=unmethylated, 1=methylated); IDH (0=wild-type, 1=mutated); P53 (0=<30%, 1=>30%); EGFR (0=negative, 1=positive)
