# Supplementary figures and images for: Deep Learning Can Differentiate IDH-Mutant from IDH-Wild GBM
Source: J Pers Med. 2021 Apr 9;11(4):290. doi: 10.3390/jpm11040290 (PMC8069494; doi:10.3390/jpm11040290)

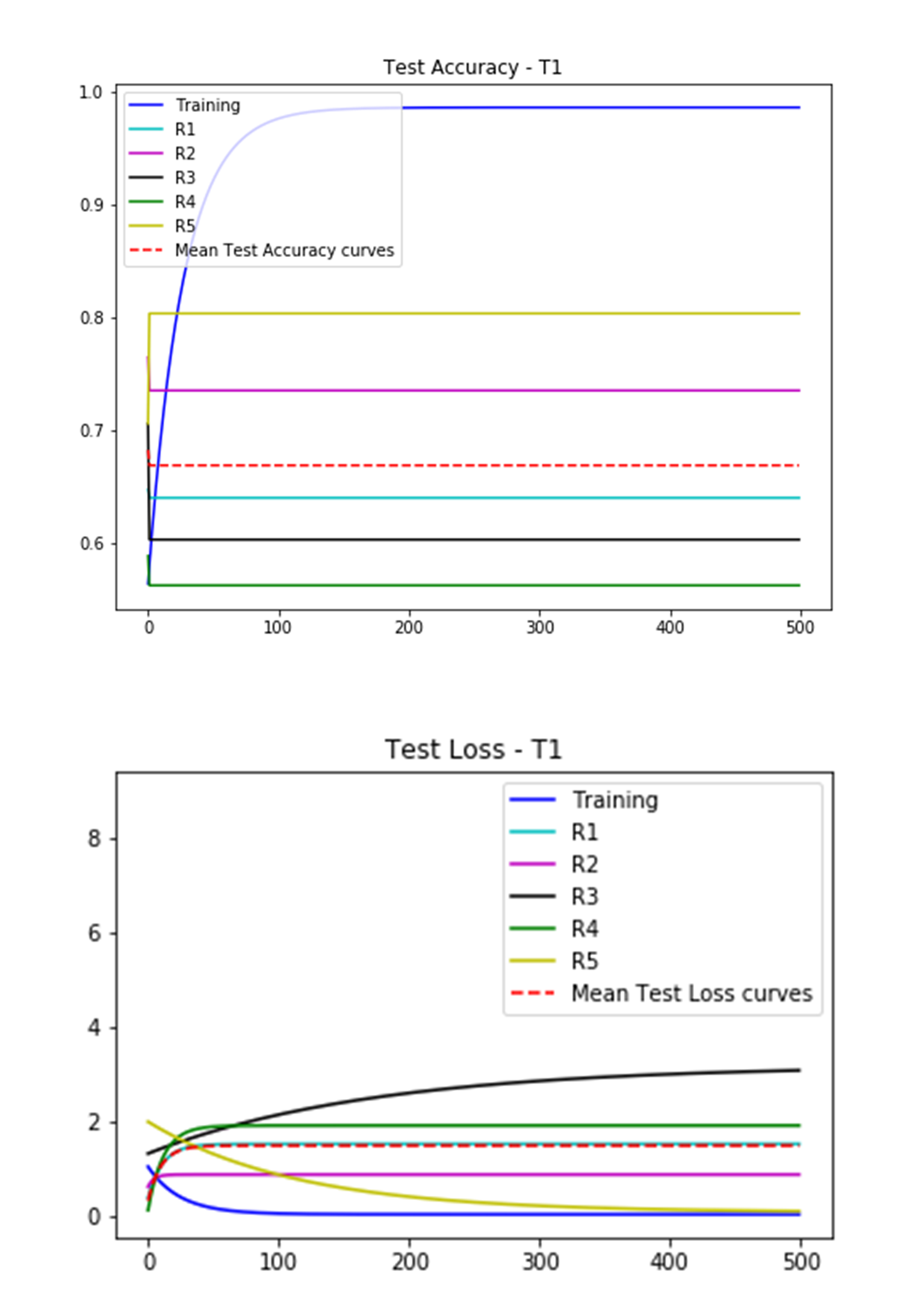

Supplement: Supplementary file 1 [file jpm-11-00290-s001.zip › Figure_1.tif]

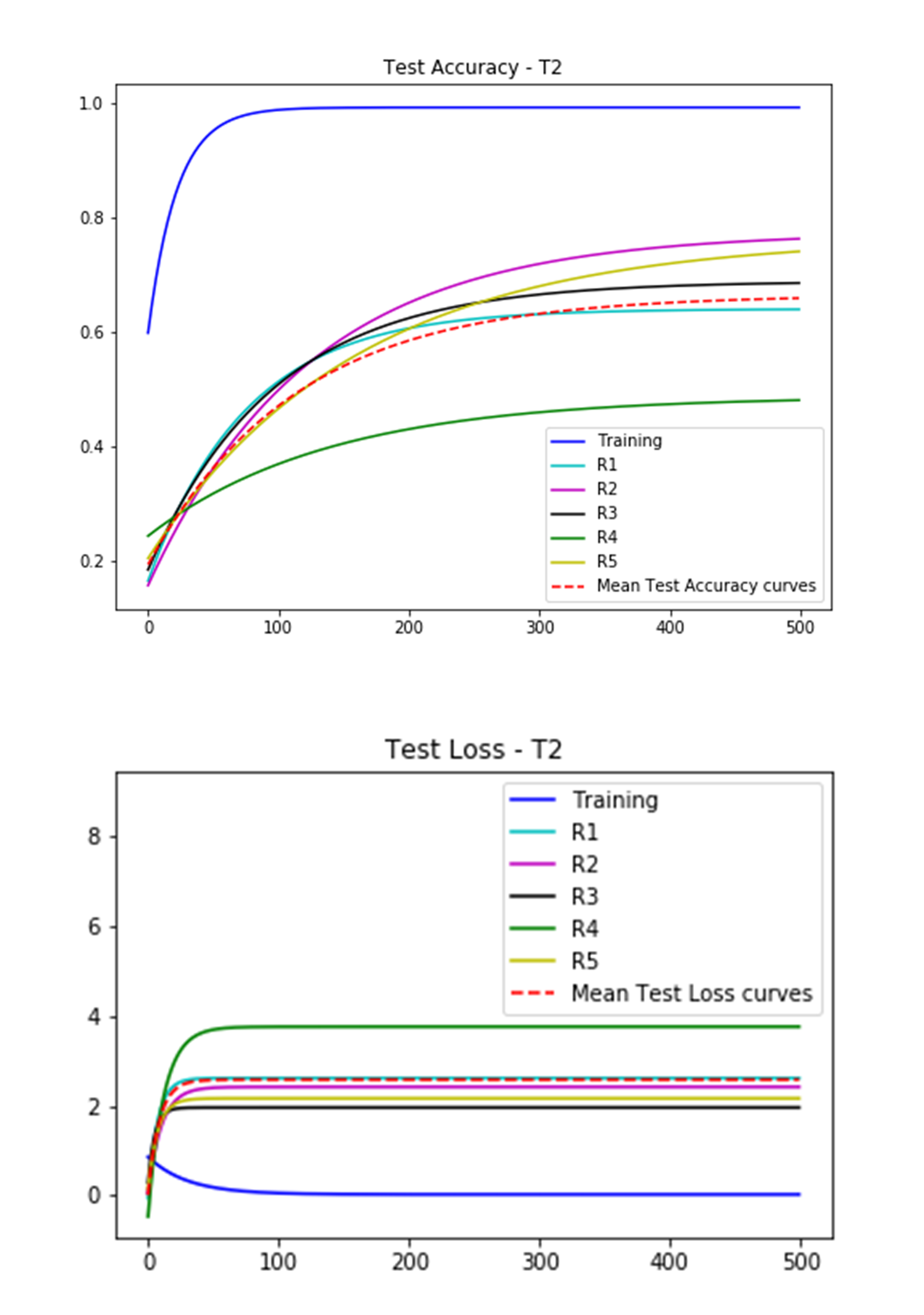

Supplement: Supplementary file 1 [file jpm-11-00290-s001.zip › Figure_2.tif]

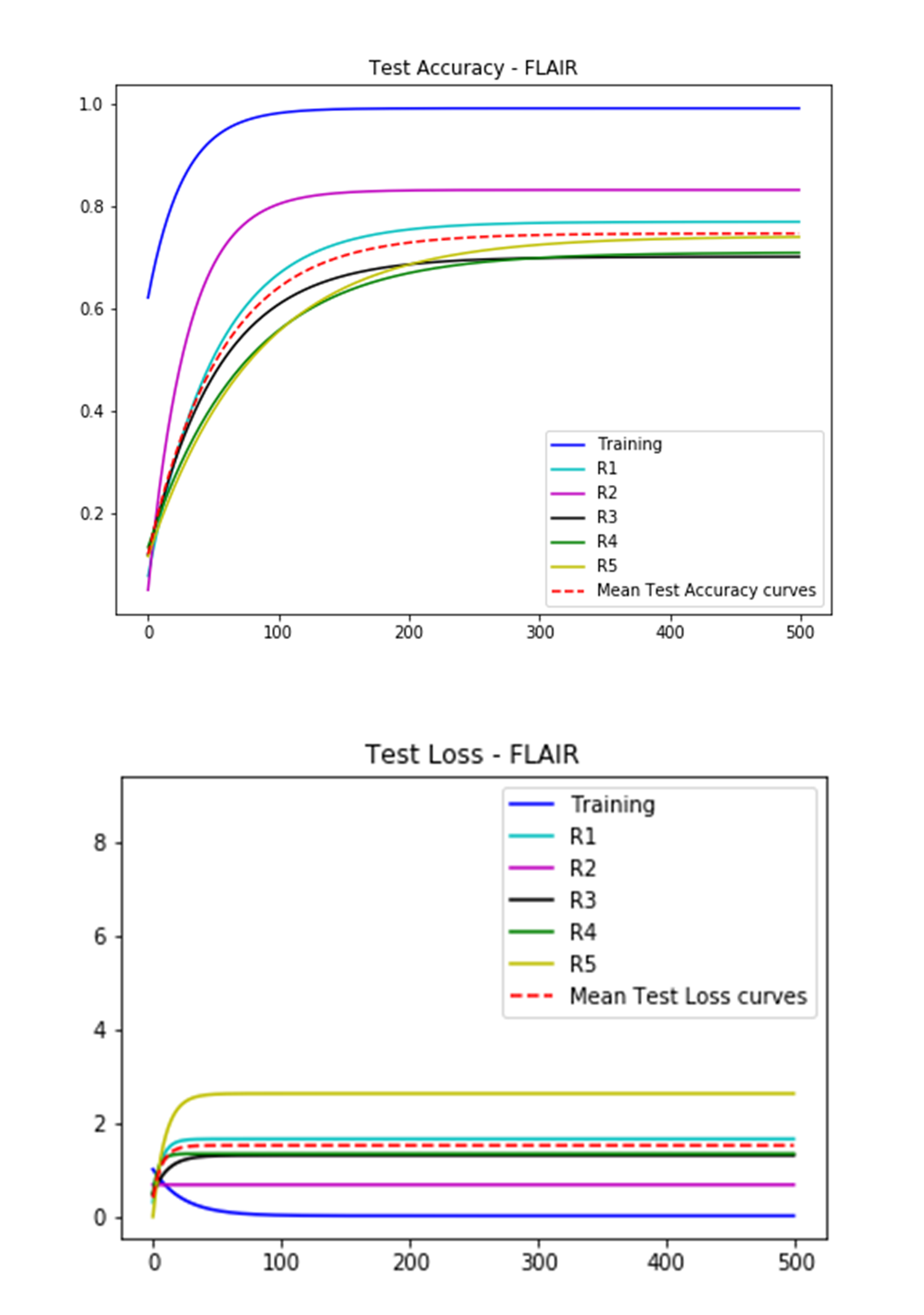

Supplement: Supplementary file 1 [file jpm-11-00290-s001.zip › Figure_3.tif]

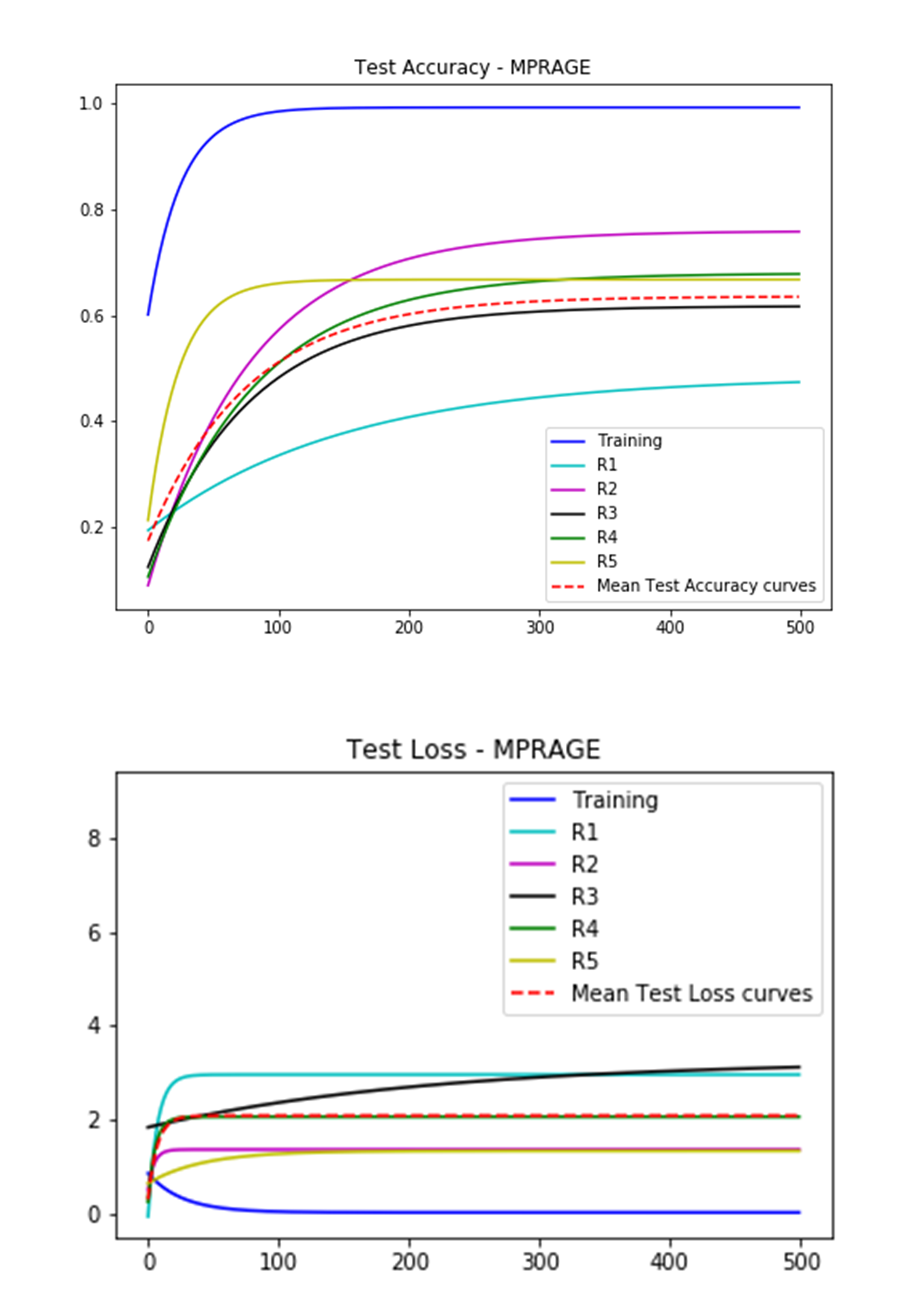

Supplement: Supplementary file 1 [file jpm-11-00290-s001.zip › Figure_4.tif]

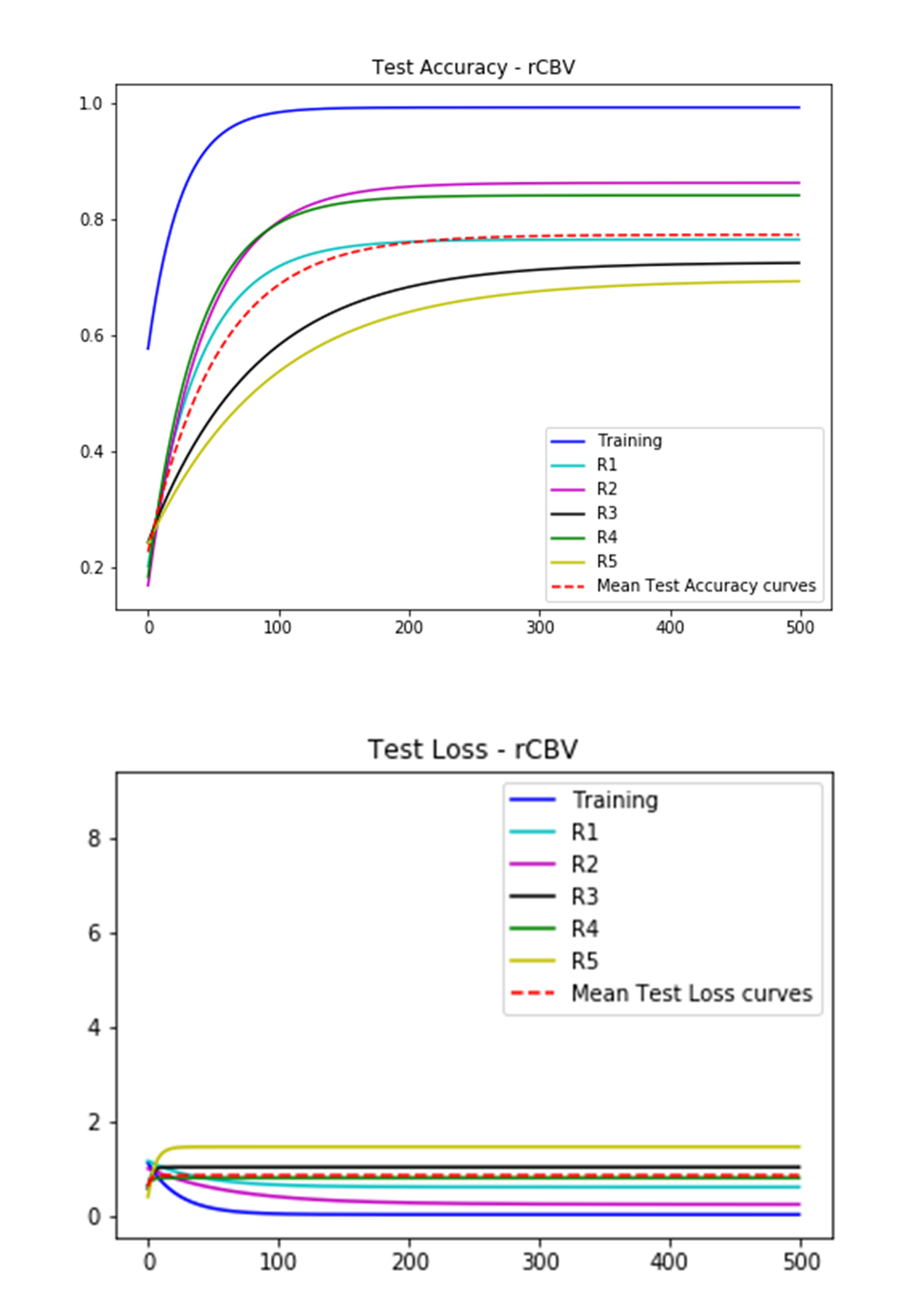

Supplement: Supplementary file 1 [file jpm-11-00290-s001.zip › Figure_5.tif]

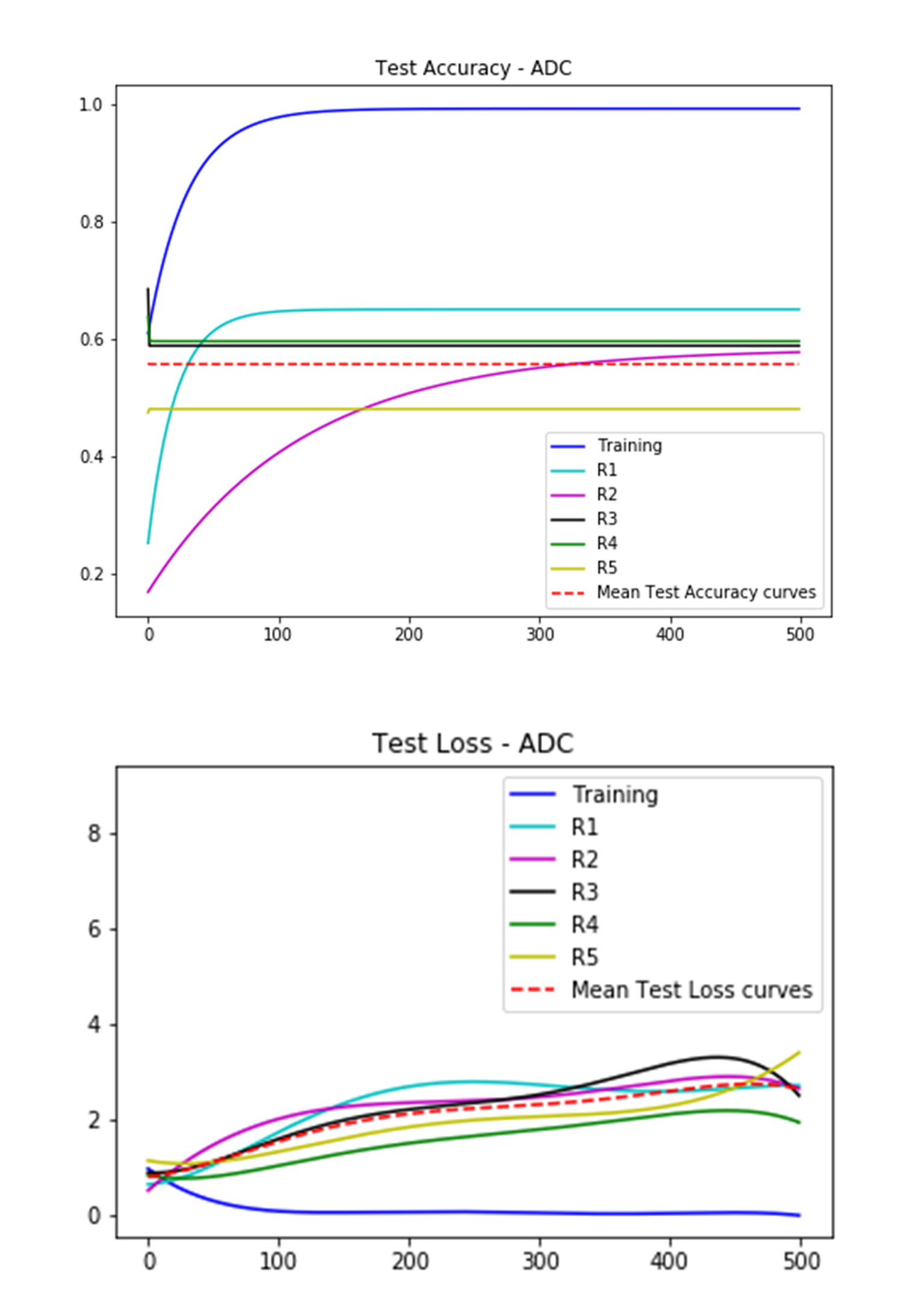

Supplement: Supplementary file 1 [file jpm-11-00290-s001.zip › Figure_6.tif]
